# A randomized controlled trial of the efficacy and safety of twice-daily saxagliptin plus metformin combination therapy in patients with type 2 diabetes and inadequate glycemic control on metformin monotherapy

**DOI:** 10.1186/1472-6823-14-17

**Published:** 2014-02-24

**Authors:** Judith L White, Patricia Buchanan, Jia Li, Robert Frederich

**Affiliations:** 1 Holston Medical Group, 105 W. Stone Drive, 37660 Kingsport, TN, USA; 2 Willamette Valley Clinical Studies, Eugene, OR, USA; 3 Bristol-Myers Squibb, Lawrenceville, NJ, USA

**Keywords:** Incretin, Dipeptidyl peptidase-4 inhibitor, Saxagliptin, Metformin, Combination therapy, Diabetes, Glycemic control, Hypoglycemia, Twice-daily

## Abstract

**Background:**

To compare the safety and efficacy of saxagliptin 2.5 mg twice daily (BID) versus placebo add-on therapy to metformin immediate release (IR) in patients with type 2 diabetes and inadequate glycemic control with metformin alone.

**Methods:**

This multicenter, 12-week, double-blind, parallel-group trial enrolled adult outpatients with type 2 diabetes (glycated hemoglobin [HbA_1c_] 7.0%–10.0%) on stable metformin IR monotherapy (≥1500 mg, BID for ≥8 weeks). Patients were randomized to double-blind saxagliptin 2.5 mg BID or placebo added on to metformin IR following a 2-week, single-blind, placebo add-on therapy lead-in period. The primary end point was the change from baseline to week 12 in HbA_1c_. Key secondary end points included change from baseline to week 12 in fasting plasma glucose (FPG) and the proportion of patients achieving HbA_1c_ <7.0% or HbA_1c_ ≤ 6.5% at week 12. Efficacy was analyzed in all patients who received randomized study drug with ≥1 postbaseline assessment. Safety was assessed in all treated patients.

**Results:**

In total, 74 patients were randomized to double-blind saxagliptin add-on therapy and 86 to placebo add-on therapy. At week 12, least-squares mean changes (95% CI) from baseline HbA_1c_ (adjusted for baseline HbA_1c_) were significantly greater (*P* = 0.006) in the saxagliptin + metformin group -0.56% (-0.74% to -0.38%) versus the placebo + metformin group -0.22% (-0.39% to -0.06%). Adjusted mean changes from baseline in FPG were numerically greater with saxagliptin versus placebo; the difference (95% CI) -9.5 mg/dL (-21.7 to 2.7) was not statistically significant (*P* = 0.12). A numerically greater proportion of patients in the saxagliptin group than the placebo group achieved HbA_1c_ < 7.0% (37.5% vs 24.2%) or HbA_1c_ ≤6.5% (24.6% vs 10.7%). There were no unexpected safety findings. Hypoglycemia occurred in 4 patients (5.4%) in the saxagliptin group and 1 patient (1.2%) in the placebo group; confirmed hypoglycemia (symptoms plus fingerstick glucose ≤50 mg/dL) occurred in 1 patient in the placebo group.

**Conclusions:**

Addition of saxagliptin 2.5 mg BID to metformin therapy in patients with type 2 diabetes and inadequate glycemic control on metformin monotherapy reduced HbA_1c_ compared with placebo added to metformin, with an adverse events profile similar to placebo and no unexpected safety findings.

**Trial registration:**

ClinicalTrials.gov NCT00885378

## Background

Type 2 diabetes is a progressive disease with multiple factors contributing to hyperglycemia—insulin resistance in muscle, liver, and adipose tissue, increased hepatic glucose production, and decreased insulin secretion [1]. Adequate glycemic control may not be possible with lifestyle interventions or medication therapy with a single agent [1,2]. Although metformin addresses some of the primary defects in insulin response (decreases hepatic glucose production and improves insulin sensitivity) [1,2] and is considered first-line pharmacologic treatment for type 2 diabetes [2,3], the American Diabetes Association and other guidelines recommend combination therapy when glycated hemoglobin (HbA_1c_) goal (<7% or ≤6.5%) [2-4] is not achieved or maintained over a 3- to 6-month period [2]. In addition, combination therapy may be appropriate as initial therapy for patients with high baseline HbA_1c_ (>7.5% or ≥9.0%) [2,4].

Because of the eventual need for combination therapy in most patients with type 2 diabetes, as well as the high prevalence of comorbidities, polypharmacy is common, and the pill burden is high [5]. Adherence to oral antihyperglycemic therapy is diminished in patients taking multiple tablets compared with those taking 1 tablet per day [6]. Cheong et al. reported significantly higher adherence to oral antidiabetic therapy in patients with type 2 diabetes using fixed-dose combination therapy versus separate-pill dual therapy; switching to fixed-dose combination therapy was associated with a 12% increase in adherence (assessed as the medication possession ratio) compared with a 5% increase for those maintaining separate-pill dual therapy [7].

Saxagliptin is a dipeptidyl peptidase-4 (DPP-4) inhibitor that inhibits degradation of the incretin hormones glucagon-like peptide-1 and glucose-dependent insulinotropic peptide, complementing the actions of metformin by reducing postprandial hyperglycemia, enhancing insulin secretion, and inhibiting paradoxical increased postprandial glucagon secretion [1,8]. Saxagliptin is approved as an adjunct to diet and exercise, as monotherapy or as an initial combination with metformin, to improve glycemic control in adults with type 2 diabetes (United States) [9,10]. It is also approved for use as combination therapy with metformin, a sulfonylurea, a thiazolidinedione, or insulin, when these agents alone, with diet and exercise, do not provide adequate glycemic control in adults with type 2 diabetes (United States and European Union) [11].

Randomized double-blind studies have evaluated the efficacy and safety of once-daily (QD) saxagliptin as add-on therapy to metformin in patients with type 2 diabetes and inadequate glycemic control receiving metformin alone [12-17]. For example, a 24-week trial in 743 patients demonstrated significant reductions in HbA_1c_ and fasting plasma glucose (FPG) with saxagliptin (2.5 mg, 5 mg, or 10 mg QD) plus metformin immediate release (IR) in divided daily doses versus placebo with metformin [12]. In another 24-week study, 286 patients with inadequate glycemic control on sub-maximal metformin IR received fixed-dose metformin IR (1500 mg/day) plus add-on saxagliptin 5 mg/day or a 2-step uptitration to a maximum dose of metformin IR (2500 mg/day) [13]. The change from baseline in HbA_1c_ at 24 weeks was similar between the 2 treatment groups. An 18-week trial in 282 patients demonstrated superior glycemic control with saxagliptin 5 mg added to extended-release (XR) metformin 1500 mg (once per evening meal) compared with increasing metformin XR to 2000 mg [14]. A 4-week trial in 93 patients revealed significantly greater reductions in 24-hour mean weighted plasma glucose in patients randomized to saxagliptin 5 mg once daily plus metformin XR (in divided daily doses) compared with those in the placebo plus metformin group [15]. In 2 randomized, double-blind studies comparing saxagliptin versus sitagliptin and versus glipizide as add-on therapy to metformin IR in divided daily doses, the improvement in glycemic control was similar with saxagliptin 5 mg and sitagliptin 100 mg over 18 weeks [16] and glipizide 5–20 mg/day (based on uptitration, as needed) over 52 weeks [17].

In addition, a 24-week randomized controlled trial with a 52-week extension evaluating saxagliptin 5 or 10 mg plus metformin IR as initial combination therapy in treatment-naive patients with type 2 diabetes demonstrated a significant and durable improvement in glycemic control with combination therapy versus either monotherapy [18,19]. Across these studies, saxagliptin plus metformin has demonstrated a favorable safety profile, with low incidences of hypoglycemia and treatment-related adverse events (AEs).

Fixed-dose combination products containing DPP-4 inhibitors and metformin are desirable to physicians as a means to potentially improve patient adherence compared with separate-pill dual therapy [20]. The current recommended dose of saxagliptin in the European Union and other markets is 5 mg QD [10]. This study investigated the safety and efficacy of saxagliptin taken as a divided dose of 2.5 mg twice daily (BID) in combination with metformin IR BID in patients with inadequate glucose control on metformin alone. With metformin IR typically given BID, a study evaluating saxagliptin using the same dosage schedule for both drugs would provide clinical data in support of a fixed-dose combination of saxagliptin with metformin IR.

## Methods

### Study design

This was a randomized (1:1), multicenter, parallel-group trial (outpatient setting) comparing saxagliptin 2.5 mg BID versus placebo BID as add-on therapy to metformin IR BID (ClinicalTrials.gov registration: NCT00885378. The study took place at 25 sites in the United States, 9 sites in Germany, 5 sites in Hungary, and 4 sites in Puerto Rico. The protocol, amendments, and patient informed consent were approved by the institutional review board(IRB)/independent ethics committee (IEC) at each site before study initiation, and the study was performed in accordance with the Declaration of Helsinki and the International Conference on Harmonisation/Good Clinical Practice. Patients provided informed consent before study participation. Each IRB/IEC was composed of a review panel that was responsible for ensuring the protection of the rights, safety, and well-being of human subjects involved in the clinical investigation and was adequately constituted to provide assurance of that protection.

### Eligibility

Patients were men and nonpregnant nonlactating women aged 18 to 78 years receiving stable metformin IR monotherapy (daily dose ≥1500 mg, given BID) for ≥8 weeks before enrollment and having inadequate glycemic control (HbA_1c_ level 7.0%–10.0% with diet and exercise). Additional inclusion criteria were a fasting C-peptide value ≥0.8 ng/mL and body mass index (BMI) ≤45.0 kg/m^2^. Patients were excluded if they had marked polydipsia and polyuria with >10% weight loss <3 months before screening, history of diabetic ketoacidosis or hyperosmolar nonketotic coma, or insulin therapy (except during short-term hospitalization or gestational diabetes) within 1 year of screening. Additional exclusion criteria included a cardiovascular event within 3 months of screening, congestive heart failure New York Heart Association class III/IV, known ejection fraction ≤40%, or history of hemoglobinopathies.

### Treatment

Patients were enrolled by investigators at each study site. At the screening visit, each patient was assigned a unique sequential subject number by an Interactive Voice Response System (IVRS), which was used for identification throughout the study. After a 2-week single-blind lead-in period with placebo BID added to metformin IR BID (with diet and exercise), patients were randomized 1:1 to 12 weeks of double-blind treatment with saxagliptin 2.5 mg BID or matching placebo BID added on to metformin IR using a blocked randomization schedule. The computer-generated randomization scheme was developed and kept by the study sponsor. Randomization was performed by calling the IVRS. Placebo tablets were identical in appearance to the saxagliptin tablets, and medication was dispensed using bottle numbers assigned by the IVRS. Titration or adjustment of blinded saxagliptin or metformin was not allowed during the study.

### Assessments

The primary end point was the change in HbA_1c_ from baseline to week 12 (or last observation carried forward [LOCF], if no week-12 value was available). Key secondary end points were FPG change from baseline to week 12, proportion of patients achieving HbA_1c_ <7.0% at week 12, and the proportion of patients achieving HbA_1c_ ≤6.5% at week 12. Other efficacy variables included the proportions of patients with 1) reductions in HbA_1c_ ≥0.5% or 2) ≥0.7%, 3) with FPG <110 mg/dL or 4) FPG <126 mg/dL, and 5) proportions requiring discontinuation for lack of glycemic control (FPG ≥270 mg/dL at 4 weeks or ≥240 mg/dL at 8 weeks). Body weight was evaluated post hoc as an exploratory end point.

Safety assessments included AEs, electrocardiograms, vital signs, physical exams, and clinical laboratory tests (ie, hematology, serum chemistry [liver function, kidney function, creatinine kinase, electrolytes, protein], and urine testing).

### Data analysis/statistics

Baseline and change from baseline efficacy assessments were analyzed in the Randomized Patients Population (those who received randomized study drug with ≥1 postbaseline assessment). The primary efficacy analysis was an analysis of covariance (ANCOVA) of the adjusted mean change in HbA_1c_ (least-squares mean adjusted for baseline HbA_1c_ value) from baseline to week 12 (or LOCF) during the double-blind period with treatment as a fixed effect and baseline HbA_1c_ as a covariate. A sample size of 152 (n = 76 per treatment group) was calculated to provide 90% power to detect an estimated 0.6% difference in HbA_1c_ with SD of 1.1%, assuming 5% of patients were unevaluable and a 2-sided α of 0.05. In addition, subgroups of HbA_1c_, gender, age and ethnicity were also analyzed by ANCOVA similar to the primary analysis.

Change from baseline to week 12 in FPG was analyzed in the same way as the primary end point. The number and proportion of patients achieving a therapeutic glycemic response (HbA_1c_ <7.0%) at week 12 LOCF was compared between groups using the methodology of Zhang et al. [21] and Tsiatis et al. [22]. A similar analysis was conducted for the proportion of patients achieving HbA_1c_ ≤6.5% at week 12. As a post hoc analysis, mean (95% CI) changes in body weight (LOCF) from baseline to week 12 were summarized by treatment group. Other efficacy end points were analyzed using descriptive statistics (n [%]).

The protocol specified the sequential testing approach to control the type I error rate at the 0.05 level. Once an end point failed to achieve statistical significance, subsequent end points in the sequence were assessed using only summary statistics, including the 95% CI. The order of testing was 1) primary end point, 2) change from baseline in FPG, 3) proportion of patients with HbA_1c_ <7.0% at week 12, and 4) proportion of patients with HbA_1c_ ≤6.5% at week 12. Statistical analyses were performed using SAS Version 8.2 or higher (SAS Institute Inc., Cary, NC).

Safety analyses were conducted using the Treated Patients Population that comprised all patients who received study drug during the double-blind treatment period. Clinical AEs were summarized using Medical Dictionary for Regulatory Activities (MedDRA) version 12.1. Events of special interest were based on a list of predefined MedDRA terms prior to database lock and unblinding of the data.

## Results

### Patients

**Figure 1:**
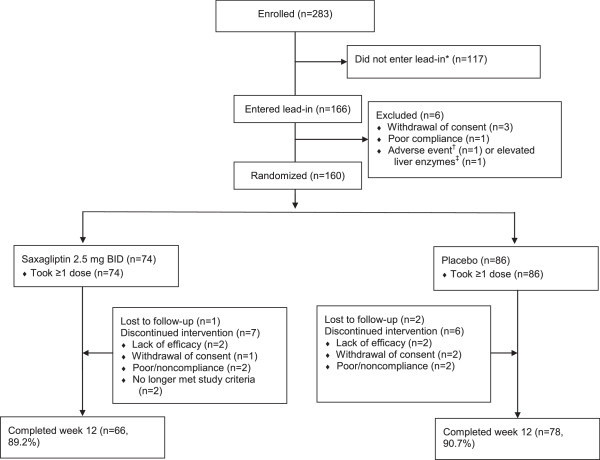
Patient disposition. ^*^Did not satisfy study inclusion and exclusion criteria. ^†^Abdominal pain secondary to partial small bowel obstruction (later classified as a serious AE, requiring hospitalization). ^‡^Values did not meet the exclusion criteria; however, the investigator chose to withdraw the patient for safety reasons.

The study was initiated on May 13, 2009 and ended on February 24, 2010 (data collection complete); recruitment took place between May 13, 2009 and November 13, 2009. A total of 160 patients were randomized to saxagliptin (n = 74) or placebo (n = 86) as add-on therapy to metformin IR and received ≥1 dose during the double-blind phase (Figure 1). All were included in the Randomized Patients Population (efficacy analysis) and the Treated Patients Population (safety analysis), and analyses were by randomized treatment group. Of those randomized and treated with drug, 144 patients (saxagliptin, n = 66; placebo, n = 78) completed 12 weeks of treatment. A similar proportion of patients in both treatment groups (saxagliptin 10.8%, placebo 9.3%) discontinued from the trial. The most common reasons for discontinuation were insufficient glycemic control (2 subjects each group: saxagliptin 2.7%; placebo 2.3%) and poor/noncompliance (2 subjects each group: saxagliptin 2.7%; placebo 2.3%). Patient characteristics are shown in Table 1; 90% were white, 40% were Hispanic or Latino, and the mean (SD) duration of type 2 diabetes was 6.0 (5.30) years.

**Table 1:** Patient demographic and baseline characteristics (randomized patients population)

**Characteristic**	**Saxagliptin + Metformin (n = 74)**	**Placebo + Metformin (n = 86)**
Men, n(%)	40 (54.1)	45 (52.3)
Mean (SD) age, y	53.9 (10.35)	56.6 (9.97)
Race, n(%)		
White	64 (86.5)	80 (93.0)
Black/African American	8 (10.8)	3 (3.5)
American Indian or Alaska native	0	1 (1.2)
Asian	2 (2.7)	2 (2.3)
Ethnicity, n(%)		
Hispanic or Latino	29 (39.2)	35 (40.7)
Not Hispanic or Latino	34 (45.9)	36 (41.9)
Not reported	11 (14.9)	15 (17.4)
BMI, kg/m^2^		
Mean (SD)	33.7 (5.94)	32.5 (6.18)
Median (range)	33.8 (19.89–44.95)	31.1 (20.68–44.64)
Mean (SD) duration of type 2 diabetes, y	5.8 (6.37)	6.2 (4.21)
Mean (SD) HbA_1c,_%	7.92 (0.961)	7.97 (0.819)
Mean (SD) FPG, mg/dL	164.9 (47.16)	161.5 (41.71)
Metformin dose (mg), n(%)		
1500– <2000	28 (37.8)	35 (40.7)
2000– <2500	38 (51.4)	45 (52.3)
≥2500	8 (10.8)	6 (7.0)

BMI = body mass index; FPG = fasting plasma glucose; HbA_1c_ = glycated hemoglobin.

### Extent of exposure

Mean (SD) duration of exposure to study drug was 80.8 (14.55) days for the saxagliptin plus metformin group and 79.7 (17.46) days for the placebo plus metformin group; >80% of patients per group continued study medication for ≥82 days.

### Efficacy

**Figure 2:**
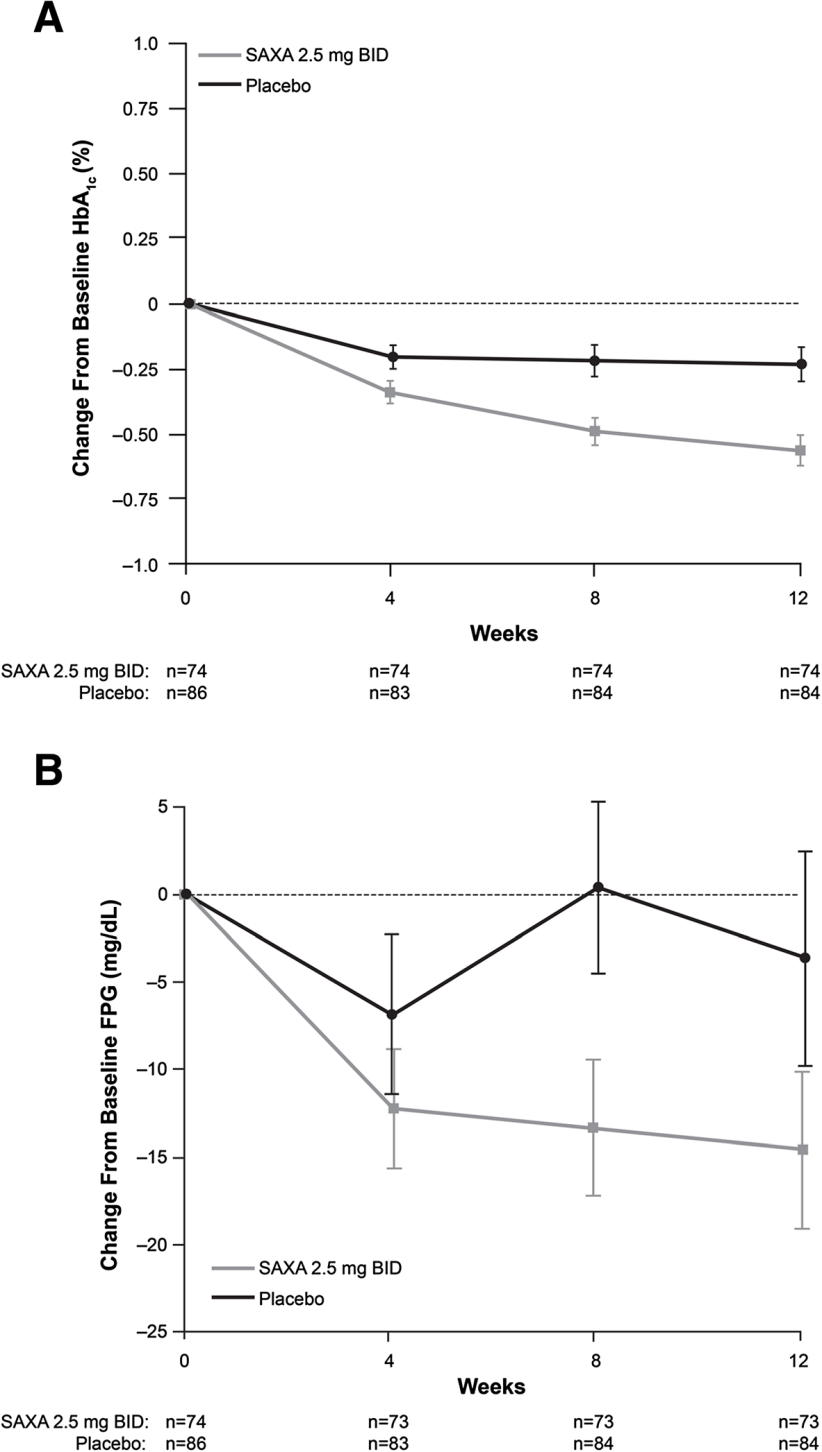
**Mean change from baseline HbA1c and fasting plasma glucose (FPG). A**. Mean (SE) change from baseline HbA_1c_ during the double-blind treatment period. BID = twice daily; HbA_1c_ = glycated hemoglobin; SAXA = saxagliptin. Mean (SE) baseline values were 7.92% (0.11) in the saxagliptin group and 7.97% (0.09) in the placebo group. **B**. Mean (SE) change from baseline fasting plasma glucose (FPG) during the double-blind treatment period. BID = twice daily; FPG = fasting plasma glucose; SAXA = saxagliptin. Mean (SE) baseline values were 164.22 mg/dL (5.51) in the saxagliptin group and 161.25 mg/dL (4.62) in the placebo group.

At week 12, adjusted mean reductions (95% CI) from baseline in HbA_1c_ were significantly greater (*P* = 0.006) in the saxagliptin plus metformin group (-0.56% [-0.74 to -0.38]) versus the placebo plus metformin group (-0.22% [-0.39 to -0.06]) (Table 2). There was a progressive decrease in HbA_1c_ to -0.56% in the saxagliptin group throughout the duration of the study (Figure 2A). There were no interactions observed for change from baseline in HbA_1c_ and treatment by baseline HbA_1c_, gender, age, or ethnicity.

**Table 2:** Primary and key secondary end points (randomized patients population)

**Efficacy variable**	**Saxagliptin + Metformin (n = 74)**	**Placebo + Metformin (n = 86)**	**Difference vs placebo**
HbA_1c_ ,%	n = 74	n = 84	
Baseline mean (SE)	7.92 (0.11)	7.97 (0.09)	
Week 12* mean (SE)	7.36 (0.13)	7.75 (0.12)	
Week 12* adjusted mean change from baseline (SE)	-0.56 (0.09)	-0.22 (0.08)	-0.34 (0.12) *P* = 0.006
FPG, mg/dL	n = 73	n = 84	
Baseline mean (SE)	164.22 (5.51)	161.25 (4.62)	
Week 12* mean (SE)	149.74 (6.18)	157.68 (4.04)	
Week 12* adjusted meanchange from baseline (SE)	-13.73 (4.51)	-4.22 (4.20)	-9.51 (6.16) *P* = 0.12
Patients achieving HbA_1c_ levels	n = 74	n = 84	
HbA_1c_ <7%, n (%)	29 (37.5)	19 (24.2)	--
HbA_1c_ ≤6.5%, n (%)	19 (24.6)	8 (10.7)	--

FPG = fasting plasma glucose; HbA_1c_ = glycated hemoglobin.

*Using last observation carried forward, applying the last postbaseline value when week 12 values were unavailable.

At week 12, adjusted mean reductions from baseline in the first secondary end point, FPG, were numerically greater (-13.73 mg/dL) in the saxagliptin plus metformin group than in the placebo plus metformin group (-4.22 mg/dL) (Figure 2B), though the difference (95% CI) of -9.5 mg/dL (-21.7 to 2.7) at week 12 was not statistically significant (*P* = 0.12) (Table 2). Accordingly, subsequent secondary end points were analyzed using only summary statistics.

The percentage of patients achieving a therapeutic glycemic response (HbA_1c_ <7%) was numerically greater with saxagliptin plus metformin versus placebo plus metformin (37.5% [29/74] vs 24.2% [19/84]). The difference (95% CI) in the proportions of patients achieving HbA_1c_ <7% versus placebo was 13.2% (1.1 to 25.4). Numerically more patients in the saxagliptin plus metformin group than the placebo plus metformin group achieved HbA_1c_ ≤6.5% (24.6% [19/74] vs 10.7% [8/84]; Table 2). The difference in the proportions of patients achieving HbA_1c_ ≤6.5% versus placebo was 13.8% (3.0 to 24.7).

### Other parameters relevant to efficacy

At week 12, a reduction in HbA_1c_ of ≥0.5% was achieved by 51.4% (38/74) and 36.9% (31/84) of patients in the saxagliptin plus metformin group and the placebo plus metformin group, respectively (difference [95% CI], 16.8% [2.3 to 31.3]). A reduction of ≥0.7% was achieved by 40.5% (30/74) and 23.8% (20/84), respectively (difference [95% CI], 14.6% [0.8 to 30.1]).

At week 12, in the saxagliptin plus metformin group and the placebo plus metformin group, 19.2% (14/73) and 3.6% (3/84), respectively, achieved FPG <110 mg/dL, and 37.0% (27/73) and 16.7% (14/84) of patients, respectively, achieved FPG <126 mg/dL.

Few patients in either group discontinued the study because of lack of glycemic control at week 4 (saxagliptin plus metformin, n = 1, 1.4%; placebo plus metformin, n = 1, 1.2%) and week 8 (saxagliptin plus metformin, n = 2, 2.7%; placebo plus metformin, n = 1, 1.2%).

Overall, there were reductions in mean body weight (LOCF) in both treatment groups. Mean change in body weight (95% CI) at week 12 was -0.32 kg (-0.97 to 0.34) for the saxagliptin plus metformin group and -0.40 kg (-0.83 to 0.02) for the placebo plus metformin group.

### Safety

During the double-blind period, the proportion of patients reporting ≥1 AE (irrespective of investigator-assessed relationship to treatment) was 25.7% (19/74) for the saxagliptin plus metformin group and 39.5% (34/86) for the placebo plus metformin group (Table 3). Treatment-related AEs as identified by the blinded investigator assessment occurred in 1 patient (1.4%) in the saxagliptin plus metformin group (nausea and dizziness; mild in severity) and 3 patients (3.5%) in the placebo plus metformin group (fatigue, increased blood creatine phosphokinase, and insomnia); no deaths occurred in either group during the 12-week double-blind treatment period (Table 3). Apart from hypoglycemia (described below), the most common AEs in the saxagliptin plus metformin group were back pain (2.7% vs 3.5% for placebo plus metformin), hypertension (2.7% vs 2.3%), dizziness (2.7% vs 0), and lymphadenopathy (2.7% vs 0; Table 4). Events with higher incidences in the placebo plus metformin group versus the saxagliptin plus metformin group were gastrointestinal (GI) AEs at the system organ class level (saxagliptin, 4.1%; placebo, 7.0%) and diarrhea (1.4% versus 3.5%). One patient in each treatment group experienced ≥1 serious AE (saxagliptin: myocardial infarction and pulmonary edema; placebo: severe back pain requiring hospitalization, owing to a lumbar strain); neither of these was considered treatment related.

**Table 3:** Patients with adverse events during the double-blind phase (treated patients population)

**n (%)**	**Saxagliptin + Metformin (n = 74)**	**Placebo + Metformin (n = 86)**
Any AE	19 (25.7)	34 (39.5)
Treatment-related AE	1 (1.4)	3 (3.5)
SAE	1 (1.4)	1 (1.2)
Treatment-related SAE	0	0
Discontinuation due to AE or SAE	0	0
Death	0	0

AE = adverse event; SAE = serious adverse event.

**Table 4:** Most common adverse events occurring in ≥2% of patients (treated patients population)

**AE, n (%)**	**Saxagliptin + Metformin (n = 74)**	**Placebo + Metformin (n = 86)**
Hypoglycemia		
All reported	4 (5.4)	1 (1.2)
Confirmed	0	1 (1.2)
Infections and infestations	5 (6.8)	11 (12.8)
Bronchitis	1 (1.4)	2 (2.3)
Nasopharyngitis	1 (1.4)	2 (2.3)
Musculoskeletal and connective tissue disorders	4 (5.4)	8 (9.3)
Back pain	2 (2.7)	3 (3.5)
Muscle spasms	1 (1.4)	3 (3.5)
Gastrointestinal disorders	3 (4.1)	6 (7.0)
Diarrhea	1 (1.4)	3 (3.5)
Blood and lymphatic system disorders	3 (4.1)	0
Lymphadenopathy	2 (2.7)	0
Vascular disorders	2 (2.7)	3 (3.5)
Hypertension	2 (2.7)	2 (2.3)
Nervous system disorders	2 (2.7)	5 (5.8)
Dizziness	2 (2.7)	0
Headache	0	3 (3.5)
Respiratory, thoracic, and mediastinal disorders	2 (2.7)	3 (3.5)
Sinus congestion	1 (1.4)	2 (2.3)
Hepatobiliary disorders	1 (1.4)	2 (2.3)
Cholecystitis	0	2 (2.3)
Psychiatric disorders	0	2 (2.3)
Insomnia	0	2 (2.3)

Confirmed hypoglycemia, defined by hypoglycemic symptoms plus a fingerstick glucose value ≤50 mg/dL, occurred in only 1 patient (placebo plus metformin group) during the double-blind period. Hypoglycemia occurred in 4 patients (5.4%) in the saxagliptin plus metformin group and 1 patient (1.2%) in the placebo plus metformin group during double-blind treatment; all were rated mild or moderate in severity. There were no events of serious hypoglycemia, and no patient discontinued the study owing to hypoglycemia during double-blind treatment. Other categories of AEs were identified as being of special interest based on findings observed in the saxagliptin nonclinical and Phase 1 and 2b programs, safety-related concerns reported for other DPP-4 inhibitors, and theoretical considerations related to the mechanism of action of DPP-4. There were 2 such AEs, both in saxagliptin-treated patients. A 63-year-old man, with hypertension, recent death of spouse, and 9-pound weight gain who quit smoking 10 days previously, suffered a non-ST-segment myocardial infarction (the only confirmed major adverse cardiovascular event [MACE] in the trial) with pulmonary edema. A 100% right coronary artery ostial occlusion with collateralization was noted, and the 80%–90% proximal left circumflex artery lesion was treated with percutaneous transluminal coronary angioplasty and a stent. Study medication was resumed and the patient completed the trial. A second patient experienced a severe AE of thrombocytopenia (baseline platelet count of 102 × 10^3^ c/μL fell to 97 × 10^3^ c/μL at the last study visit). The patient was asymptomatic, and the event was deemed not related to treatment. There were no reports of other AEs of special interest, including skin disorders, lymphopenia, thrombocytopenia, localized edema, hypersensitivity, fractures, or pancreatitis. There was no clinically meaningful drug effect on any other laboratory safety parameter, including hematologic, liver, renal, electrolytes, protein, and musculoskeletal parameters.

## Discussion

In the study reported here, saxagliptin 2.5 mg BID in combination with metformin IR BID for 12 weeks significantly reduced HbA_1c_ in patients with type 2 diabetes with inadequate glycemic control with metformin alone. Saxagliptin had an AE profile similar to placebo and no unexpected safety findings.

Our findings add to an existing literature demonstrating that the addition of saxagliptin to metformin therapy improves glucose control in patients with type 2 diabetes. Across 3 previous trials of saxagliptin 5 mg QD as add-on therapy in patients with inadequate glycemic control on metformin IR monotherapy, adjusted mean changes from baseline HbA_1c_ were -0.52% (18-week trial of saxagliptin versus sitagliptin as add-on therapy to metformin) [16], -0.69% (24-week trial of saxagliptin versus placebo as add-on therapy to metformin) [12], and -0.74% (52-week trial of saxagliptin versus glipizide as add-on therapy to metformin) [17]; and between-group differences in adjusted mean change from baseline HbA_1c_ (95% CI) were 0.09% (-0.01% to 0.20%), -0.83% (-1.02% to -0.63%), and 0.06% (-0.05% to 0.16%), respectively. In these same 3 studies, adjusted mean changes from baseline FPG were -10.8, -22.0, and -9 mg/dL, respectively. In the current study, adjusted mean change from baseline HbA_1c_ was -0.56% with saxagliptin 2.5 mg BID in combination with metformin IR BID, and the mean (SE) between-group difference was -0.34% (0.12%). To further place these findings in context, the UK Prospective Diabetes Study Group demonstrated a 37% decrease in risk of microvascular complications for each 1% reduction in HbA_1c_[23]. Although HbA_1c_ is considered an appropriate surrogate marker for microvascular outcomes [24], macrovascular risk reduction was not an end point in the current study.

In a previous trial of saxagliptin plus metformin IR as initial combination therapy, significantly greater effectiveness versus the individual monotherapies was demonstrated for reduction of HbA_1c_ (2.5% reduction vs 1.7% and 2.0% for saxagliptin and metformin alone, respectively) and FPG (reduction of 60 mg/dL vs 31 mg/dL and 47 mg/dL for saxagliptin and metformin alone, respectively) [18].

In the current study, reductions in placebo-adjusted FPG, though of similar magnitude with the statistically significant reductions in FPG with saxagliptin add-on therapy in other studies, did not reach statistical significance. This precluded formal assessment of the proportions of patients achieving HbA_1c_ targets. However, the proportions of patients achieving HbA_1c_ <7% or ≤6.5% were numerically greater for the saxagliptin plus metformin group compared with the placebo plus metformin group and consistent with previous short-term studies of saxagliptin added to metformin or existing oral antihyperglycemic therapy (HbA_1c_ <7% range, 22.8%–43.5%) [12,14,25,26]. Because lowering HbA_1c_ to ≤7% has been demonstrated to reduce microvascular complications [24], this end point provides further context regarding the clinical relevance of glycemic improvement in this study.

The low rate of hypoglycemic events and small change in body weight observed in this BID dosing study are consistent with those observed in previous studies [12,15-18]. GI AEs were not exacerbated with respect to incidence or severity by the addition of saxagliptin. Overall, the present study provides reassurance of the safety and tolerability of saxagliptin plus metformin when both are given as divided BID doses.

We acknowledge several limitations of this study, including the 12-week timeframe and the relatively small sample size. As there is known interindividual variability in red blood cell lifespan [27], 12 weeks may not be sufficient to demonstrate the full potential for HbA_1c_ lowering in a study population. The absence of statistical significance of the first prespecified secondary end point of FPG precluded further sequential assessment for statistical significance of other key secondary end points. Because FPG has greater biologic and analytic variability than HbA_1c_[28], this study may not have been powered to detect changes in FPG. However, other categorical measures of glycemic control were numerically improved with saxagliptin given twice daily, supporting the demonstration of improved glycemic control compared with placebo when added to metformin. Inclusion criteria necessarily limited the heterogeneity of the study population, which could affect generalizability. Although there are ethical concerns regarding placebo-controlled designs, this approach was important for initial evaluation of this new regimen of saxagliptin administration in order to evaluate the potential bias of clinical study participation. The trial was limited to 12 weeks in duration, and patients were discontinued from treatment and withdrawn from the study if they had lack of glycemic control during the double-blind treatment period at weeks 4 and 8. An active-controlled study may provide important further information about the clinical utility of this regimen.

## Conclusions

In patients with type 2 diabetes and inadequate glycemic control on metformin monotherapy, the addition of saxagliptin, a medication with a mechanism of action complementary to metformin [29], significantly reduced HbA_1c_ compared with the addition of placebo. The present study demonstrates that saxagliptin 2.5 mg BID added to ongoing metformin IR BID was effective in reducing HbA_1c_ levels compared with placebo, with a similar AE profile and no unexpected safety findings.

## Competing interests

JL and RF are employees of Bristol-Myers Squibb. JW serves on a speaker’s bureau for Pfizer and has been a clinical investigator for Bristol-Myers Squibb, Pfizer, Johnson & Johnson, Boehringer Ingelheim, Gilead, Regeneron, Amgen, Novo Nordisk, Orexigen, Lilly, and Merck. PB has no competing interests.

## Authors’ contributions

JW and PB were primary investigators on the study and participated in the collection and interpretation of the data. JL had primary responsibility for the protocol and participated in the statistical analysis and interpretation of the data. RF participated in the study design and data interpretation. All authors participated in the manuscript outline/content development, provided critical review of all drafts, read and approved the final manuscript, and made the decision to submit to *BMC Endocrine Disorders*.

## Pre-publication history

The pre-publication history for this paper can be accessed here:

http://www.biomedcentral.com/1472-6823/14/17/prepub

## References

[B1] Zinman B Initial combination therapy for type 2 diabetes mellitus: is it ready for prime time? Am J Med 2011 124 1 Suppl S19 S34 21194577 10.1016/j.amjmed.2010.11.003

[B2] Inzucchi SE Bergenstal RM Buse JB Diamant M Ferrannini E Nauck M Peters AL Tsapas A Wender R Matthews DR Management of hyperglycemia in type 2 diabetes: a patient-centered approach: position statement of the American Diabetes Association (ADA) and the European Association for the Study of Diabetes (EASD) Diabetes Care 2012 35 6 1364 1379 10.2337/dc12-0413 22517736 PMC3357214

[B3] Global guideline for type 2 diabetes http://www.staff.ncl.ac.uk/philip.home/IDF%20GGT2D.pdf

[B4] Handelsman Y Mechanick JI Blonde L Grunberger G Bloomgarden ZT Bray GA Dagogo-Jack S Davidson JA Einhorn D Ganda O Garber AJ Hirsch IB Horton ES Ismail-Beigi F Jellinger PS Jones KL Jovanovič L Lebovitz H Levy P Moghissi ET Orzeck EA Vinik AI Wyne KL American Association of Clinical Endocrinologists medical guidelines for clinical practice for developing a diabetes mellitus comprehensive care plan Endocr Pract 2011 17 2 Suppl 1 53 21474420 10.4158/ep.17.s2.1

[B5] Blonde L San Juan ZT Fixed-dose combinations for treatment of type 2 diabetes mellitus Adv Ther 2012 29 1 1 13 10.1007/s12325-011-0094-1 22271157

[B6] Donnan PT MacDonald TM Morris AD Adherence to prescribed oral hypoglycaemic medication in a population of patients with type 2 diabetes: a retrospective cohort study Diabet Med 2002 19 4 279 284 10.1046/j.1464-5491.2002.00689.x 11942998

[B7] Cheong C Barner JC Lawson KA Johnsrud MT Patient adherence and reimbursement amount for antidiabetic fixed-dose combination products compared with dual therapy among Texas Medicaid recipients Clin Ther 2008 30 10 1893 1907 10.1016/j.clinthera.2008.10.003 19014846

[B8] Davidson JA Incorporating incretin-based therapies into clinical practice: differences between glucagon-like peptide 1 receptor agonists and dipeptidyl peptidase 4 inhibitors Mayo Clin Proc 2010 85 12 Suppl S27 S37 21106865 10.4065/mcp.2010.0469 PMC2996165

[B9] KOMBIGLYZE XR (saxagliptin and metformin hydrochloride extended-release) tablets http://packageinserts.bms.com/pi/pi_kombiglyze_xr.pdf

[B10] Onglyza^®^ (saxagliptin) tablets http://packageinserts.bms.com/pi/pi_onglyza.pdf

[B11] Onglyza. Summary of product characteristics http://www.ema.europa.eu/docs/en_GB/document_library/EPAR_-_Product_Information/human/001039/WC500044316.pdf

[B12] DeFronzo RA Hissa MN Garber AJ Luiz Gross J Yuyan Duan R Ravichandran S Chen RS The efficacy and safety of saxagliptin when added to metformin therapy in patients with inadequately controlled type 2 diabetes with metformin alone Diabetes Care 2009 32 9 1649 1655 10.2337/dc08-1984 19478198 PMC2732156

[B13] Hermans MP Delibasi T Farmer I Lohm L Maheux P Piatti P Malvolti E Jorgens S Charbonnel B Effects of saxagliptin added to sub-maximal doses of metformin compared with uptitration of metformin in type 2 diabetes: the PROMPT study Curr Med Res Opin 2012 28 10 1635 1645 10.1185/03007995.2012.735646 23020253

[B14] Fonseca V Zhu T Karyekar C Hirshberg B Adding saxagliptin to extended-release metformin vs. uptitrating metformin dosage Diabetes Obes Metab 2012 14 4 365 371 10.1111/j.1463-1326.2011.01553.x 22192246

[B15] Stenlof K Raz I Neutel J Ravichandran S Berglind N Chen R Saxagliptin and metformin XR combination therapy provides glycemic control over 24 hours in patients with T2DM inadequately controlled with metformin Curr Med Res Opin 2010 26 10 2355 2363 10.1185/03007995.2010.511090 20804445

[B16] Scheen AJ Charpentier G Ostgren CJ Hellqvist A Gause-Nilsson I Efficacy and safety of saxagliptin in combination with metformin compared with sitagliptin in combination with metformin in adult patients with type 2 diabetes mellitus Diabetes Metab Res Rev 2010 26 7 540 549 10.1002/dmrr.1114 20824678

[B17] Göke B Gallwitz B Eriksson J Hellqvist A Gause-Nilsson I Saxagliptin is non-inferior to glipizide in patients with type 2 diabetes mellitus inadequately controlled on metformin alone: a 52-week randomised controlled trial Int J Clin Pract 2010 64 12 1619 1631 10.1111/j.1742-1241.2010.02510.x 20846286

[B18] Jadzinsky M Pfutzner A Paz-Pacheco E Xu Z Allen E Chen R Saxagliptin given in combination with metformin as initial therapy improves glycaemic control in patients with type 2 diabetes compared with either monotherapy: a randomized controlled trial Diabetes Obes Metab 2009 11 6 611 622 10.1111/j.1463-1326.2009.01056.x 19515181

[B19] Pfützner A Paz-Pacheco E Allen E Frederich B Chen R for the CV181039 Investigators Initial combination therapy with saxagliptin and metformin provides sustained glycaemic control and is well tolerated for up to 76 weeks Diabetes Obes Metab 2011 13 6 567 576 10.1111/j.1463-1326.2011.01385.x 21342412

[B20] Benford M Milligan G Pike J Anderson P Piercy J Fermer S Fixed-dose combination antidiabetic therapy: real-world factors associated with prescribing choices and relationship with patient satisfaction and compliance Adv Ther 2012 29 1 26 40 10.1007/s12325-011-0096-z 22246944

[B21] Zhang M Tsiatis AA Davidian M Improving efficiency of inferences in randomized clinical trials using auxiliary covariates Biometrics 2008 64 3 707 715 10.1111/j.1541-0420.2007.00976.x 18190618 PMC2574960

[B22] Tsiatis AA Davidian M Zhang M Lu X Covariate adjustment for two-sample treatment comparisons in randomized clinical trials: a principled yet flexible approach Stat Med 2008 27 23 4658 4677 10.1002/sim.3113 17960577 PMC2562926

[B23] Stratton IM Adler AI Neil HA Matthews DR Manley SE Cull CA Hadden D Turner RC Holman RR Association of glycaemia with macrovascular and microvascular complications of type 2 diabetes (UKPDS 35): prospective observational study BMJ 2000 321 7258 405 412 10.1136/bmj.321.7258.405 10938048 PMC27454

[B24] American Diabetes Association Standards of medical care in diabetes--2013 Diabetes Care 2013 36 1 Suppl S11 S66 23264422 10.2337/dc13-S011 PMC3537269

[B25] Chacra AR Tan GH Apanovitch A Ravichandran S List J Chen R Saxagliptin added to a submaximal dose of sulphonylurea improves glycaemic control compared with uptitration of sulphonylurea in patients with type 2 diabetes: a randomised controlled trial Int J Clin Pract 2009 63 9 1395 1406 10.1111/j.1742-1241.2009.02143.x 19614786 PMC2779994

[B26] Hollander P Li J Allen E Chen R CV181-013 Investigators Saxagliptin added to a thiazolidinedione improves glycemic control in patients with type 2 diabetes and inadequate control on thiazolidinedione alone J Clin Endocrinol Metab 2009 94 12 4810 4819 10.1210/jc.2009-0550 19864452

[B27] Cohen RM Franco RS Khera PK Smith EP Lindsell CJ Ciraolo PJ Palascak MB Joiner CH Red cell life span heterogeneity in hematologically normal people is sufficient to alter HbA1c Blood 2008 112 10 4284 4291 10.1182/blood-2008-04-154112 18694998 PMC2581997

[B28] Bonora E Tuomilehto J The pros and cons of diagnosing diabetes with A1C Diabetes Care 2011 34 2 Suppl S184 S190 21525453 10.2337/dc11-s216 PMC3632159

[B29] Freeman JS Managing hyperglycemia in patients with type 2 diabetes mellitus: rationale for the use of dipeptidyl peptidase-4 inhibitors in combination with other oral antidiabetic drugs J Am Osteopath Assoc 2010 110 9 528 537 20876838

